# Effect of Sequential or Active Choice for Colorectal Cancer Screening Outreach: A Randomized Clinical Trial

**DOI:** 10.1001/jamanetworkopen.2019.10305

**Published:** 2019-08-30

**Authors:** Shivan J. Mehta, Vikranth Induru, David Santos, Catherine Reitz, Timothy McAuliffe, Charles Orellana, Kevin G. Volpp, David A. Asch, Chyke A. Doubeni

**Affiliations:** 1 Department of Medicine, Perelman School of Medicine, University of Pennsylvania, Philadelphia; 2 Center for Health Incentives and Behavioral Economics, Leonard Davis Institute of Health Economics, University of Pennsylvania, Philadelphia; 3 Center for Health Care Innovation, University of Pennsylvania, Philadelphia; 4 Department of Family Medicine and Community Health, Perelman School of Medicine, University of Pennsylvania, Philadelphia; 5 Clinical Care Associates, University of Pennsylvania, Philadelphia; 6 Center for Health Equity Research and Promotion, Philadelphia VA Medical Center, Philadelphia, Pennsylvania

## Abstract

**Importance:**

Colonoscopy and fecal immunochemical testing (FIT) are considered top-tier tests for colorectal cancer (CRC) screening. Behavioral economic insights about “choice architecture” suggest that participation could be influenced by how people are presented test options.

**Objective:**

To investigate response rates for offering colonoscopy only compared with sequential choice (colonoscopy and then FIT) or active choice (colonoscopy or FIT) through mailed outreach.

**Design, Setting, and Participants:**

Three-arm pragmatic randomized clinical trial conducted between November 14, 2017, and May 14, 2018. The setting was primary care practices at an academic health system. Patients aged 50 to 74 years with at least 2 primary care visits in the 2-year preenrollment period were included if they were eligible but not up to date on CRC screening.

**Interventions:**

Eligible patients received mailed outreach about CRC screening. Equal numbers of eligible patients were randomly assigned to 3 outreach groups to receive mailings about CRC screening with the following options: (1) direct phone number to call for scheduling colonoscopy (colonoscopy only), (2) direct phone number to call for colonoscopy and a mailed FIT kit if no response within 4 weeks (sequential choice), or (3) direct phone number to call for colonoscopy and a mailed FIT kit offered at the same time (active choice).

**Main Outcomes and Measures:**

The primary outcome was CRC screening completion (FIT or colonoscopy) within 4 months of initial outreach. The secondary outcomes were CRC screening completion within 6 months of outreach and the choice of colonoscopy as a screening test.

**Results:**

In total, 438 patients were included in the intent-to-treat analysis, with a median age of 56 years (interquartile range, 52-63 years); 55.0% were women. At 4 months, the CRC screening completion rates were 14.4% (95% CI, 8.7%-20.1%) in the colonoscopy-only arm, 17.1% (95% CI, 11.0%-23.2%) in the sequential choice arm, and 19.9% (95% CI, 13.4%-26.4%) in the active choice arm. Neither choice arm achieved a screening rate statistically greater than that in the colonoscopy-alone arm. Among those who completed CRC screening at 4 months, 90.5% (95% CI, 78.0%-103.0%) chose colonoscopy in the colonoscopy-only arm, which was significantly higher than the 52.0% (95% CI, 32.4%-71.6%; *P* = .005) and 37.9% (95% CI, 20.2%-55.6%; *P* < .001) in the sequential choice and active choice arms, respectively.

**Conclusions and Relevance:**

There was no significant increase in CRC screening when offering sequential or active choice, but there was a lower rate of colonoscopy in the choice arms than in the colonoscopy-only arm. Subtle changes in sequencing or defaults can alter patient decision making related to preventive health.

**Trial Registration:**

ClinicalTrials.gov identifier: NCT03246438

## Key Points

**Question:**

Does the offering of choice between colonoscopy and fecal immunochemical testing (FIT) change participation in screening outreach?

**Findings:**

In this 3-arm pragmatic randomized clinical trial of 438 patients, there was no statistically significant increase in screening when offering the choice of FIT compared with colonoscopy only, but fewer patients selected colonoscopy in the choice arms.

**Meaning:**

Offering the choice of FIT in a colonoscopy outreach program did not substantially increase screening participation, but the framing of choice altered patient decision making.

## Teaser

This 3-arm pragmatic randomized clinical trial investigates response rates for offering colonoscopy only compared with sequential choice (colonoscopy and then fecal immunochemical testing) or active choice (colonoscopy or fecal immunochemical testing) through mailed outreach.

## Introduction

Screening is an effective preventive intervention for reducing the risk of death from colorectal cancer (CRC), but uptake is suboptimal despite considerable efforts.^1,2,3,4,5^ Colonoscopy and fecal immunochemical testing (FIT) are both considered top-tier tests according to recent guidelines.^6,7,8^ Mailing FIT directly to patients’ homes has been shown to boost CRC screening rates,^9,10,11,12,13^ but it requires annual outreach to be effective, while colonoscopy may be needed only every 10 years. Guidelines also suggest offering multiple options together (colonoscopy or FIT) or sequentially (colonoscopy and then FIT among those who decline),^6^ but there is limited evidence of how the differing approaches to offering choice in screening alter uptake and pattern of testing.^14,15,16,17,18^

Behavioral economic insights about “choice architecture” suggest that screening participation could be influenced by how the choices are presented to patients.^19,20,21,22,23^ Conventional thinking implies that giving patients a choice of screening options allows them to align screening with their preferences. This would suggest that greater choice is always preferable for patients. However, more choices may also overwhelm patients and reduce participation and satisfaction through choice overload.^24,25,26^ In addition, sequential choice may increase the completion of colonoscopy vs FIT by leveraging status quo bias and framing colonoscopy as the default option.^19,27,28^ Colonoscopy’s infrequent screening interval offers advantage over yearly testing with FIT, but FIT offers simplicity and convenience of completing screening at home without the need for bowel preparation. In this study, we investigated response rates for offering colonoscopy only compared with sequential choice (colonoscopy and then FIT) or active choice (colonoscopy or FIT) through mailed outreach.

## Methods

### Study Design

**Figure 1.**
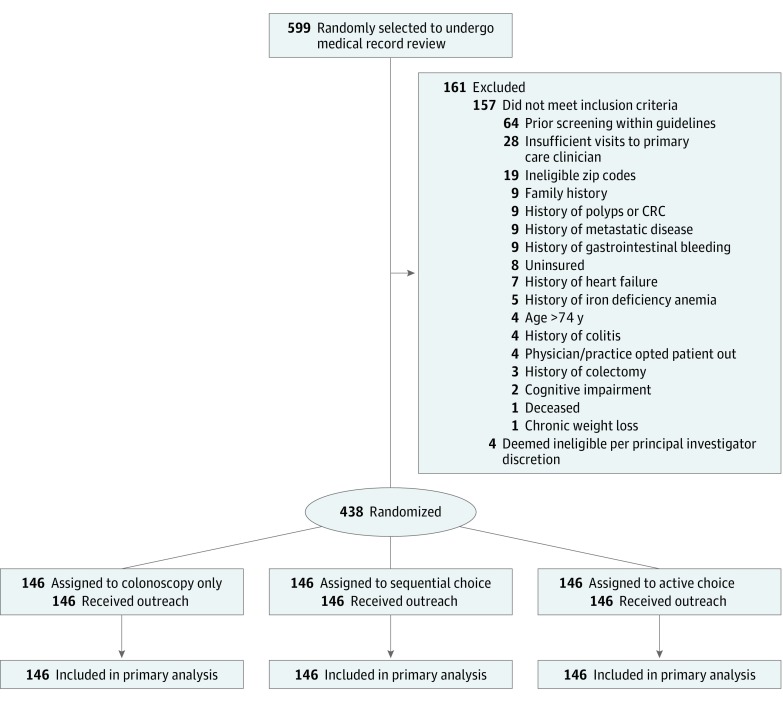
CONSORT Diagram of Randomized Clinical Trial to Increase Rates of Colorectal Cancer Screening Some patients were excluded for multiple reasons. CONSORT indicates Consolidated Standards of Reporting Trials; CRC, colorectal cancer.

This was a 3-arm pragmatic randomized clinical trial of mailed outreach to determine the effectiveness of offering different choices of colonoscopy only or with FIT for CRC screening. We compared the following approaches: (1) direct phone number to call for scheduling colonoscopy (colonoscopy only), (2) direct phone number to call for colonoscopy and a mailed FIT kit if no response within 4 weeks (sequential choice), or (3) direct phone number to call for colonoscopy and a mailed FIT kit offered together (active choice). The study was approved by the institutional review board at the University of Pennsylvania. A waiver of informed consent was obtained because the study posed minimal risk to patients and could not have been practicably carried out without the waiver.^29^ The trial protocol and statistical analysis plan are available in Supplement 1. This study followed the Consolidated Standards of Reporting Trials (CONSORT) reporting guideline, including the checklist and diagram to track participants during the enrollment and trial procedures (Figure 1).

### Study Population

The study included patients at 2 primary care practices at the University of Pennsylvania serving a sociodemographically diverse population in the Philadelphia region. Patients were identified through automated data extraction from the electronic health record (EHR) in August 2017. We included patients aged 50 to 74 years with at least 2 primary care visits to the clinic in the 2-year preenrollment period who were overdue for CRC screening and had a home zip code within the Philadelphia-Wilmington-Camden Metropolitan Statistical Area. Overdue was defined as not having completed colonoscopy within the past 10 years, flexible sigmoidoscopy within the past 5 years, or stool testing within the past year. We excluded patients with a personal or family history of CRC, colonic polyps, hereditary nonpolyposis colorectal cancer syndrome, familial adenomatous polyposis syndrome, other gastrointestinal cancer, gastrointestinal bleeding, iron-deficiency anemia, inflammatory bowel disease, other colitis, or history of colectomy. Patients were also excluded if they had a diagnosis of end-stage renal disease, cirrhosis, metastatic cancer, congestive heart failure, or dementia because such conditions may compromise life expectancy and outweigh the benefits of screening.

Medical record review for all patients was performed by research staff after the automated data pull to confirm eligibility from August to October 2017. For all included patients, primary care clinicians (physicians or nurse practitioners) were provided an opportunity to remove any of their patients from participation through opt-out messaging.

### Interventions

Eligible patients were randomized in a 1:1:1 allocation ratio using a computerized random number generator. All patients received a letter from their primary care clinician describing the benefits of CRC screening and indicating that they were overdue according to health records. Those in the colonoscopy-only arm received a phone number to call for direct scheduling of colonoscopy through the call center. If they did not schedule or complete colonoscopy in 4 weeks, they received a follow-up letter describing the same information about colonoscopy scheduling. Those in the sequential choice arm received a phone number to call for direct scheduling of colonoscopy. If they did not schedule or complete colonoscopy in 4 weeks, they received a follow-up letter describing the colonoscopy scheduling information and an enclosed FIT kit. The FIT kit included a sample collection tube, instructions for use, a phone number to call with questions, and a stamped envelope to mail the collected sample to the laboratory at no cost to the patient. Those in the active choice arm received a phone number to call for direct scheduling of colonoscopy and an enclosed FIT kit with materials described above. If they did not respond in 4 weeks, they received a follow-up letter describing colonoscopy scheduling information and information about the previously sent FIT kit.

The patients who scheduled colonoscopy received the standard information about preparation instructions, and those who completed the procedure received follow-up from the endoscopist about the results. All FIT results were sent to the patient’s primary care clinician, and patients with negative results received a letter. For patients with positive results, research staff contacted the primary care clinician directly to coordinate follow-up diagnostic colonoscopy; in such cases, patients received both a phone call and a follow-up letter. The investigators were masked to patient data and randomization, but the research staff were not masked because they were administering the interventions.

### Postintervention Interviews

After the follow-up time frame was completed, 30 participants from each arm were randomly selected to complete a follow-up semistructured interview about their experience with CRC screening, the outreach materials, and choice of testing. Research staff made up to 3 attempts to contact the selected participants.

### Study Outcomes

The primary outcome was CRC screening completion (FIT or colonoscopy) within 4 months of initial outreach. The secondary outcomes were CRC screening completion within 6 months of outreach and the choice of colonoscopy as a screening test among those who completed screening at 4 and 6 months. The CRC screening completion rate was any documented evidence of CRC screening in the EHR, including those screened through study processes or routine care. We also tracked the results of screening colonoscopy and the outcomes of patients with positive FIT, including receipt of colonoscopy and the findings during colonoscopy.

Data were obtained from the EHR through automated data extraction and medical record review by research staff. Race/ethnicity data based on self-reported data in the EHR to evaluate response rate because many studies have shown racial/ethnic differences in screening participation. Household income was estimated using the American Community Survey 2012-2016 five-year estimates data for median income by zip code of residence.

### Statistical Analysis

Based on a prior study^30^ using the direct colonoscopy scheduling process, we estimated a 5% base return rate for the colonoscopy-only arm. Enrollment of 423 participants allowed us to detect an 11% increase in response rate (comparing each choice arm with the control arm) using a 2-sided χ^2^ test with 80% power and a type I error rate of 0.025, accounting for 3 pairwise comparisons with Bonferroni correction (*P* value of .05 / 2 = .025). We report response rates as proportions with 95% CIs. Comparisons between arms were performed using the test of proportions between each choice arm and the control arm for the 4-month and 6-month response rates and the choice of colonoscopy. We also performed subgroup analyses for the primary outcome by sex, race/ethnicity, and practice site. All analyses were performed using statistical software (Stata, version 15.0; StataCorp).

## Results

### Patient Characteristics

A total of 599 potentially eligible patients were identified through automated data extraction; 438 of these patients were included after medical record review and randomly assigned to the 3 study arms (Figure 1). The median age was 56 years (interquartile range [IQR], 52-63 years); 55.0% were women. Approximately 76.9% (337 of 438) had commercial insurance, and 15.9% (70 of 438) were insured by Medicare. About 49% (215 of 438) of patients were non-Hispanic white, and 36.1% (158 of 438) were non-Hispanic black (Table 1). The median household income was $84 868 (IQR, $32 873-$111 892). One patient in the active choice arm died during the study follow-up period, but all patients were included in the intent-to-treat analysis. The intervention was conducted between November 14, 2017, and May 14, 2018, when 6-month follow-up was completed for all randomized participants.

**Table 1.** Demographic Characteristics by Study Arm Assignment

Variable	No. (%)		
	Colonoscopy Only (n = 146)	Sequential Choice (n = 146)	Active Choice (n = 146)
Female	82 (56.2)	81 (55.5)	78 (53.4)
Age, median (IQR), y	57 (53-63)	57 (54-63)	55 (52-61)
Race/ethnicity^a^			
Non-Hispanic white	74 (50.7)	66 (45.2)	75 (51.4)
Non-Hispanic black	50 (34.2)	52 (36.3)	55 (37.7)
Other	10 (6.8)	10 (6.8)	4 (2.7)
Unknown	12 (8.2)	18 (12.3)	12 (8.2)
Insurance type			
Commercial	120 (82.2)	107 (73.3)	110 (75.3)
Medicare	18 (12.3)	25 (17.1)	27 (18.5)
Medicaid	8 (5.5)	13 (8.9)	9 (6.2)
Uninsured	0	1 (0.7)	0
Household income, median (IQR), $^b^	84 868 (32 873-102 842)	84 868 (32 873-101 577)	88 594 (37 819-113 441)

Abbreviation: IQR, interquartile range.

^a^ There were 10 people of Asian race/ethnicity in the colonoscopy-only arm, 8 in the sequential choice arm, and 4 in the active choice arm. There were 1 Hispanic individual and 1 Hawaiian/Pacific Islander individual in sequential choice.

^b^ Based on American Community Survey 2012-2016 five-year estimates data.

### CRC Screening Completion

At 4 months, the CRC screening completion rates were 14.4% (95% CI, 8.7%-20.1%) in the colonoscopy-only arm, 17.1% (95% CI, 11.0%-23.2%) in the sequential choice arm, and 19.9% (95% CI, 13.4%-26.4%) in the active choice arm (Table 2). The screening rate in neither choice arm was statistically greater than that in the colonoscopy-only arm at 4 months. At 6 months, the completion rate was 18.5% (95% CI, 12.2%-24.8%) in the colonoscopy-only arm, 19.2% (95% CI, 12.8%-25.6%) in the sequential choice arm, and 23.3% (95% CI, 16.4%-30.2%) in the active choice arm. There was also no statistically significant difference by arm in screening completion at 6 months.

**Table 2.** Screening Participation

Variable	No. (%) [95% CI]		*P* Value	No. (%) [95% CI]	*P* Value
	Colonoscopy Only (n = 146)	Sequential Choice (n = 146)		Active Choice (n = 146)	
**CRC Screening Within 4 mo**					
FIT	2 (1.4) [−0.5 to 3.1]	13 (8.9) [4.3 to 13.5]	NA	19 (13.0) [7.5 to 18.5]	NA
Colonoscopy	19 (13.0) [7.5 to 18.5]	13 (8.9) [4.3 to 13.5]	NA	11 (7.5) [3.2 to 11.8]	NA
Any CRC screening	21 (14.4) [8.7 to 20.1]	25 (17.1) [11.0 to 23.2]^a^	.53	29 (19.9) [13.4 to 26.4]^b^	.21
Choice of colonoscopy, % (95% CI)	90.5 (78.0 to 103.0)	52.0 (32.4 to 71.6)^c^	.005	37.9 (20.2 to 55.6)^c^	<.001
**CRC Screening Within 6 mo**					
FIT	4 (2.7) [0.1 to 5.3]	13 (8.9) [4.3 to 13.5]	NA	20 (13.7) [8.1 to 19.3]	NA
Colonoscopy	23 (15.8) [9.9 to 21.7]	16 (11.0) [5.9 to 16.1]	NA	14 (9.6) [4.8 to 14.4]	NA
Any CRC screening	27 (18.5) [12.2 to 24.8]	28 (19.2) [12.8 to 25.6]^a^	.88	34 (23.3) [16.4 to 30.2]^b^^,^^d^	.31
Choice of colonoscopy, % (95% CI)	85.2 (71.8 to 98.6)	57.1 (38.8 to 75.4)^c^	.02	41.2 (24.7 to 57.7)^c^	<.001

Abbreviations: CRC, colorectal cancer; FIT, fecal immunochemical testing; NA, not applicable.

^a^ One patient completed both screening colonoscopy and FIT in the sequential choice arm at 4 months.

^b^ One patient completed both screening colonoscopy and FIT in the active choice arm at 4 months.

^c^ *P* value of .05 / 2 = .025 was the threshold for statistical significance with Bonferroni correction to account for 2 pairwise comparisons.

^d^ One patient completed computed tomographic colonography at 6 months, which counted for any CRC screening.

Among the 53 patients who completed screening colonoscopy, 19 (35.8%) had adenomas (1 had an adenoma with high-grade dysplasia), and 1 (1.9%) was diagnosed as having colon cancer. Among the 37 patients who completed FIT, 2 (5.4%) were found to be positive, and both followed up with diagnostic colonoscopy. One of those patients had no polyps, and the other had a tubular adenoma with high-grade dysplasia. When evaluating by arm and including the results of screening and diagnostic colonoscopy after FIT, patients in the colonoscopy-only arm had 8 adenomas, those in the sequential choice arm had 8 adenomas, and those in the active choice arm had 4 adenomas and 1 cancer.

We also analyzed CRC screening response rates at 4 months by arm and overall among women, men, non-Hispanic white individuals, non-Hispanic black individuals, and the 2 primary care practices. Women had a response rate of 9.8% (8 of 82) in colonoscopy only, 17.3% (14 of 81) in sequential choice, and 21.8% (17 of 78) in active choice (*P* = .04). Otherwise, there were no differences in response rate by arm and overall among the other subgroups.

### CRC Screening Choice

**Figure 2.**
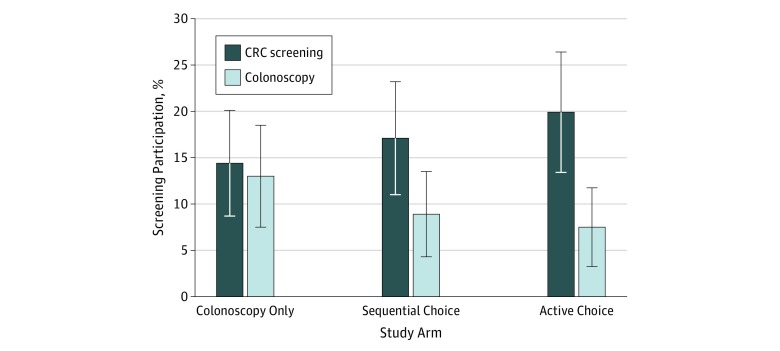
CRC Screening Completion at 4 Months by Study Arm Error bars are 95% CI. CRC indicates colorectal cancer.

Among those who completed CRC screening at 4 months, 90.5% (95% CI, 78.0%-103.0%) chose colonoscopy in the colonoscopy-only arm, which was significantly higher than the 52.0% (95% CI, 32.4%-71.6%; *P* = .005) and 37.9% (95% CI, 20.2%-55.6%; *P* < .001) who chose colonoscopy in the sequential choice and active choice arms, respectively (Figure 2 and Table 2). Among those who completed CRC screening at 6 months, 85.2% (95% CI, 71.8%-98.6%) chose colonoscopy in the colonoscopy-only arm, 57.1% (95% CI, 38.8%-75.4%; *P* = .02) in the sequential choice arm, and 41.2% (95% CI, 24.7%-57.7%; *P* < .001) in the active choice arm.

### Postintervention Interviews

Of the 438 patients in the study, 90 (20.5%) were called to complete the postintervention interview, and 26 (28.9%) agreed to participate. Fifteen of the 26 patients (57.7%) reported receiving the mailed outreach invitation. Fourteen of the 26 patients (53.8%) would prefer to get screened by FIT in the future, with reasons including the ease of completion at home and that it does not require preparation or sedation. Eight of the 26 patients (30.8%) would prefer colonoscopy because it is required only every 10 years and it is recommended by a physician. Among 10 patients who completed the interview in the colonoscopy-only arm, 4 (40.0%) preferred FIT, and 5 (50.0%) preferred colonoscopy (the 10th patient did not have a preference). Among 9 interview participants in the sequential choice arm, 5 (55.6%) preferred FIT and 1 (11.1%) preferred colonoscopy (the other 3 patients did not have a preference between the 2 tests). Among 7 patients who completed the interview in the active choice arm, 5 (71.4%) preferred FIT and 2 (28.6%) preferred colonoscopy.

## Discussion

In this study of mailed CRC screening outreach, we found that adding the choice of FIT did not result in a statistically significant increase in overall completion rate compared with offering colonoscopy only. Although the overall rate did not change substantially, the framing of choice between colonoscopy and FIT altered the patient selection of screening test. Offering the choice of FIT reduced selection of colonoscopy, and active choice resulted in an even lower rate of colonoscopy compared with sequential choice.

These findings add to the literature exploring the role of choice in CRC screening promotion activities. One study^14^ that evaluated screening in a clinic setting showed that offering the choice of colonoscopy or fecal testing resulted in higher initial rates than offering colonoscopy only, but a follow-up study^17^ demonstrated that choice did not have greater uptake than colonoscopy at 3 years. Offering FIT sequentially to patients who did not respond to a population-based flexible sigmoidoscopy program increased screening participation.^18^ Similar to our results, 2 studies^15,16^ that evaluated different choices of testing through mailed outreach did not find increased uptake.

There are a few possible reasons why we did not find substantially higher rates of overall screening when offering the choice of FIT in colonoscopy outreach. First, the additional choice may have increased choice overload by expecting patients to choose between options with multiple different characteristics and considerations. In other contexts, when consumers are offered too many choices of items, they decrease overall consumption and are less satisfied.^24,25,26^ In this case, even 1 additional choice may have reduced overall participation, perhaps because this is a prevention activity with immediate costs and distant future benefits. Although we did not directly compare offering FIT alone in this trial, 2 recent studies^20,31^ at different primary care practices in the same health system offering similar mailed FIT outreach language showed response rates of 29.1% at 3 months and 28.9% at 6 months, respectively, which was higher than what we found for both choice options that included the same mailed FIT materials. Another study^11^ demonstrated a 16.1% increase in CRC screening rate in response to mailed FIT kits compared with colonoscopy outreach alone. One would expect that offering colonoscopy in addition to mailed FIT should have a response rate no worse than that in mailed FIT alone.

Second, we offered the choice of screening in a mailed setting, which did not allow for patients to discuss the comparative risks and benefits of different screening options with a clinician. This is important to consider because health systems often rely on proactive population-based outreach programs. Third, our study was conducted in an insured population where colonoscopy is the main screening modality offered during office visits, which may explain greater participation in colonoscopy in all our study arms. Fourth, our sample size limited our ability to detect smaller increases in uptake by the choice arms that may be clinically important, and there is the possibility that choice may be associated with a modest increase in participation. However, given that colonoscopy is typically performed up to every 10 years, while FIT requires outreach every year, the higher rate of overall screening would need to be balanced with lower choice of colonoscopy.

In addition to the main findings about overall completion of screening, we also found that the framing of choice dramatically changed the decision between colonoscopy or FIT. In the colonoscopy-only arm, 90.5% of participating patients chose colonoscopy, while 37.9% chose colonoscopy in the active choice arm. Making colonoscopy the only or first choice implies to patients that it is the default option. In situations of complex decision making where patients may not have strong preferences, they will often be subject to status quo bias because the other option requires them to overcome inertia and opt out.^19,22,27,28,32^ A prior study^20^ showed that switching from opt-in to opt-out framing increased participation in mailed FIT from 10% to 29%. Our findings demonstrate that preference for the default option can also apply to choices between different screening options. One limitation to the sequential choice arm was that FIT was mailed 1 month after initial outreach if colonoscopy was not scheduled or completed. There is the possibility that waiting longer than 1 month could have allowed for greater participation in colonoscopy, which may be evaluated in future studies.

### Strengths and Limitations

The strengths of this study are the prospective design and individual-level randomization. This was a pragmatic trial embedded in routine clinical operations, so the results reflect how different choices may affect patient behavior in real-world settings. The study population includes both urban and suburban clinical practices with a diverse population, which is similar to many health systems across the country. We also addressed an important clinical question, which is reflected in guidelines and clinical practice, but has not been evaluated to our knowledge in population health outreach programs.

There are also some limitations to this study. First, we did not have an arm that focused on mailed FIT only, although there are 2 concurrent studies^20,31^ in the same health system that provide information about response rates. Second, we only focused on choice for mailed CRC screening outreach, so we were not able to fully address the differences in screening options. Future studies could explore choice architecture in the clinic setting, in which screening decision making can be observed directly. Third, our population included insured patients in an academic health system, so the results may not translate to other underserved settings. Fourth, we did not track outcomes after 6 months, and our time between colonoscopy and FIT for sequential choice was only 4 weeks. Longer-term studies could offer repeated reminders about colonoscopy before mailing an FIT kit in a sequential choice strategy to increase the choice of colonoscopy. Fifth, although we conducted medical record review for enrollment and outcomes, we may have missed CRC screening that occurred outside our health system. If there was differential capture of screening activities outside the health system between colonoscopy and FIT, we would likely miss more colonoscopies occurring at other facilities, which would have resulted in less of a difference in uptake between colonoscopy only and the choice arms.

## Conclusions

Our results show that subtle changes in sequencing or defaults can alter patient decision making related to preventive health. This has implications for CRC screening, as well as for many other clinical areas where clinicians have to guide patients on choices with many characteristics and trade-offs.

## Notes

Supplement 1. **Trial Protocol and Statistical Analysis Plan**

Supplement 2. **Data Sharing Statement**

## References

[1] Siegel RL, Miller KD, Jemal A Cancer statistics, 2018. CA Cancer J Clin. 2018;68(1):-. doi:10.3322/caac.21442 29313949

[2] Mandel JS, Bond JH, Church TR, Reducing mortality from colorectal cancer by screening for fecal occult blood: Minnesota Colon Cancer Control Study. N Engl J Med. 1993;328(19):1365-1371. doi:10.1056/NEJM199305133281901 8474513

[3] Schoen RE, Pinsky PF, Weissfeld JL, ; PLCO Project Team Colorectal-cancer incidence and mortality with screening flexible sigmoidoscopy. N Engl J Med. 2012;366(25):2345-2357. doi:10.1056/NEJMoa1114635 22612596 PMC3641846

[4] Doubeni CA, Corley DA, Quinn VP, Effectiveness of screening colonoscopy in reducing the risk of death from right and left colon cancer: a large community-based study. Gut. 2018;67(2):291-298. doi:10.1136/gutjnl-2016-312712 27733426 PMC5868294

[5] White A, Thompson TD, White MC, Cancer screening test use: United States, 2015. MMWR Morb Mortal Wkly Rep. 2017;66(8):201-206. doi:10.15585/mmwr.mm6608a1 28253225 PMC5657895

[6] Rex DK, Boland CR, Dominitz JA, Colorectal cancer screening: recommendations for physicians and patients from the U.S. Multi-Society Task Force on Colorectal Cancer. Am J Gastroenterol. 2017;112(7):1016-1030. doi:10.1038/ajg.2017.174 28555630

[7] Bibbins-Domingo K, Grossman DC, Curry SJ, ; US Preventive Services Task Force Screening for colorectal cancer: US Preventive Services Task Force recommendation statement [published corrections appear in *JAMA*. 2016;316(5):545 and 2017;317(21):2239]. JAMA. 2016;315(23):2564-2575. 27304597 10.1001/jama.2016.5989

[8] Wolf AMD, Fontham ETH, Church TR, Colorectal cancer screening for average-risk adults: 2018 guideline update from the American Cancer Society. CA Cancer J Clin. 2018;68(4):250-281. doi:10.3322/caac.21457 29846947

[9] Green BB, Wang CY, Anderson ML, An automated intervention with stepped increases in support to increase uptake of colorectal cancer screening: a randomized trial. Ann Intern Med. 2013;158(5 pt 1):301-311. doi:10.7326/0003-4819-158-5-201303050-00002 23460053 PMC3953144

[10] Mehta SJ, Jensen CD, Quinn VP, Race/ethnicity and adoption of a population health management approach to colorectal cancer screening in a community-based healthcare system. J Gen Intern Med. 2016;31(11):1323-1330. doi:10.1007/s11606-016-3792-1 27412426 PMC5071288

[11] Gupta S, Halm EA, Rockey DC, Comparative effectiveness of fecal immunochemical test outreach, colonoscopy outreach, and usual care for boosting colorectal cancer screening among the underserved: a randomized clinical trial. JAMA Intern Med. 2013;173(18):1725-1732. 23921906 10.1001/jamainternmed.2013.9294 PMC5228201

[12] Myers RE, Sifri R, Hyslop T, A randomized controlled trial of the impact of targeted and tailored interventions on colorectal cancer screening. Cancer. 2007;110(9):2083-2091. doi:10.1002/cncr.23022 17893869

[13] Dougherty MK, Brenner AT, Crockett SD, Evaluation of interventions intended to increase colorectal cancer screening rates in the United States: a systematic review and meta-analysis. JAMA Intern Med. 2018;178(12):1645-1658. doi:10.1001/jamainternmed.2018.4637 30326005 PMC6583619

[14] Inadomi JM, Vijan S, Janz NK, Adherence to colorectal cancer screening: a randomized clinical trial of competing strategies. Arch Intern Med. 2012;172(7):575-582. doi:10.1001/archinternmed.2012.332 22493463 PMC3360917

[15] Segnan N, Senore C, Andreoni B, ; SCORE2 Working Group–Italy Randomized trial of different screening strategies for colorectal cancer: patient response and detection rates. J Natl Cancer Inst. 2005;97(5):347-357. doi:10.1093/jnci/dji050 15741571

[16] Multicentre Australian Colorectal-Neoplasia Screening (MACS) Group A comparison of colorectal neoplasia screening tests: a multicentre community-based study of the impact of consumer choice. Med J Aust. 2006;184(11):546-550. 16768659 10.5694/j.1326-5377.2006.tb00377.x

[17] Liang PS, Wheat CL, Abhat A, Adherence to competing strategies for colorectal cancer screening over 3 years. Am J Gastroenterol. 2016;111(1):105-114. doi:10.1038/ajg.2015.367 26526080 PMC4887132

[18] Senore C, Ederle A, Benazzato L, Offering people a choice for colorectal cancer screening. Gut. 2013;62(5):735-740. doi:10.1136/gutjnl-2011-301013 22442162

[19] Halpern SD, Ubel PA, Asch DA Harnessing the power of default options to improve health care. N Engl J Med. 2007;357(13):1340-1344. doi:10.1056/NEJMsb071595 17898105

[20] Mehta SJ, Khan T, Guerra C, A randomized controlled trial of opt-in versus opt-out colorectal cancer screening outreach. Am J Gastroenterol. 2018;113(12):1848-1854. doi:10.1038/s41395-018-0151-3 29925915 PMC6768589

[21] Loewenstein G, Brennan T, Volpp KG Asymmetric paternalism to improve health behaviors. JAMA. 2007;298(20):2415-2417. doi:10.1001/jama.298.20.2415 18042920

[22] Mehta SJ, Asch DA How to help gastroenterology patients help themselves: leveraging insights from behavioral economics. Clin Gastroenterol Hepatol. 2014;12(5):711-714. doi:10.1016/j.cgh.2014.02.022 24746170

[23] Tversky A, Kahneman D The framing of decisions and the psychology of choice. Science. 1981;211(4481):453-458. doi:10.1126/science.7455683 7455683

[24] Iyengar SS, Lepper MR When choice is demotivating: can one desire too much of a good thing? J Pers Soc Psychol. 2000;79(6):995-1006. doi:10.1037/0022-3514.79.6.995 11138768

[25] Schwartz B The tyranny of choice. Sci Am. 2004;290(4):70-75. doi:10.1038/scientificamerican0404-70 15045756

[26] Vohs KD, Baumeister RF, Schmeichel BJ, Twenge JM, Nelson NM, Tice DM Making choices impairs subsequent self-control: a limited-resource account of decision making, self-regulation, and active initiative. J Pers Soc Psychol. 2008;94(5):883-898. doi:10.1037/0022-3514.94.5.883 18444745

[27] Johnson EJ, Goldstein D Medicine: do defaults save lives? Science. 2003;302(5649):1338-1339. doi:10.1126/science.1091721 14631022

[28] Chapman GB, Li M, Colby H, Yoon H Opting in vs opting out of influenza vaccination. JAMA. 2010;304(1):43-44. doi:10.1001/jama.2010.892 20606147

[29] Asch DA, Ziolek TA, Mehta SJ Misdirections in informed consent: impediments to health care innovation. N Engl J Med. 2017;377(15):1412-1414. doi:10.1056/NEJMp1707991 29020586

[30] Mehta SJ, Feingold J, Vandertuyn M, Active choice and financial incentives to increase rates of screening colonoscopy: a randomized controlled trial. Gastroenterology. 2017;153(5):1227-1229.e2. doi:10.1053/j.gastro.2017.07.015 28734830 PMC5669820

[31] Mehta SJ, Pepe RS, Gabler NB, Effect of financial incentives on patient use of mailed colorectal cancer screening tests: a randomized clinical trial. JAMA Netw Open. 2019;2(3):e191156. doi:10.1001/jamanetworkopen.2019.1156 30901053 PMC6583304

[32] Yudin MH, Moravac C, Shah RR Influence of an “opt-out” test strategy and patient factors on human immunodeficiency virus screening in pregnancy. Obstet Gynecol. 2007;110(1):81-86. doi:10.1097/01.AOG.0000267497.39041.06 17601900

