# Supplementary material for: Effect of Sequential or Active Choice for Colorectal Cancer Screening Outreach: A Randomized Clinical Trial
Source: JAMA Netw Open. 2019 Aug 30;2(8):e1910305. doi: 10.1001/jamanetworkopen.2019.10305 (PMC6724166; doi:10.1001/jamanetworkopen.2019.10305)
Supplement: Supplement 1. — Trial Protocol and Statistical Analysis Plan [file jamanetwopen-2-e1910305-s001.pdf]

Supplement

Choice Architecture and Colorectal Cancer Screening Outreach

This supplement provides additional information about the work. It contains the following items:

Initial Protocol …… 1

Final Protocol …… 13

Summary of protocol changes …… 24

Original statistical analysis plan …… 25

Final statistical analysis plan …… 26

Summary of statistical analysis plan changes …… 27

Appendix A: Final Survey Instrument …… 28

Appendix B: Mailed Outreach Language …… 30

Initial Protocol

Abstract
A 3-arm randomized trial assessing whether the rate of completion of colorectal cancer screening is increased when patients receive a sequential choice in screening options (colonoscopy followed by Fecal Immunochemical Testing (FIT)) or an active choice (FIT or colonoscopy offered together) versus colonoscopy alone. The targeted population is patients within the University City and Valley Forge Community Care Associates (CCA) practices at the University of Pennsylvania Health System.

Study Instruments
The primary endpoint being evaluated is the rate of participation in colorectal cancer screening between the two intervention arms (Sequential Choice, Active Choice) versus the control arm (colonoscopy only). The FIT is a well validated tool for colorectal cancer screening and is one of the screening modalities recommended by the USPSTF. The control arm will be sent a letter inviting them to schedule screening colonoscopy directly through the Gastroenterology Call Center. If not scheduled within 4 weeks, subjects will receive a mailed reminder to call to schedule. The Sequential Choice arm will be sent the same initial outreach as the control arm. If not scheduled within 4 weeks, subjects will receive a mailed reminder including the call center number as well as the option to complete a FIT kit mailed along with the reminder. The Active Choice arm of the study will be sent a letter offering the choice of Colonoscopy or completion of the FIT kit included with the initial mailing. If subjects in this group have not either scheduled colonoscopy nor completed FIT within 4 weeks, they will receive a mailed reminder to call to schedule colonoscopy or to complete the original mailed FIT.

A sub-sample of 90 subjects will be called to complete a questionnaire over the phone 4 months after initial outreach was mailed. The subjects will be asked to confirm their eligibility (e.g. that they had not had CRC screening within the USPSTF CRC screening guidelines) and provide additional demographic and socioeconomic information so that we can better understand what populations, if any, may have differential response rates. We will also ask them about their perception of the impact and design of CRC screening outreach. Demographic and socioeconomic questions are modified from demographic questions on the Behavioral Risk Factor Surveillance System survey, administered by the Centers for Disease Control and Prevention. See attached sub-sample questionnaire.

Group Modifications
For subjects in the Control (Colonoscopy only) arm of the study, the post outreach phone questionnaires will not include questions regarding mailed FIT.

Method for Assigning Subjects to Groups
Subjects will be randomly assigned Study ID numbers and then randomized to one of three study arms stratified by the two practice locations (University City and Valley Forge) using a computer-generated randomization algorithm. The research coordinator will record the randomization assignments on a master list which will be maintained by the research coordinator on a password protected computer in a locked office. The research coordinator and research assistants will assemble the mailings based on this master list.

Administration of Surveys and/or Process
90 subjects will be randomly selected for the questionnaire. We anticipate the post outreach questionnaires to take 10 minutes to complete over the phone. The research staff will make no more than three attempts to speak directly with the subject. Based on a previous project where we reached about 50% of patients via phone call, we anticipate reaching approximately 45 subjects (15 in each arm) to complete this sub-sample questionnaire.

Administration of Surveys
All subjects will complete a baseline and post-intervention survey. The baseline survey will collect basic demographics and baseline medication adherence and blood pressure monitoring frequency and be conducted over the phone after the participant has been consented. The post-intervention survey will collect similar adherence and monitoring information, as well as qualitative data regarding patient perceptions about the interventions. The post-intervention survey will be completed at the in-person 4 month visit. These surveys should take no more than 15 minutes to complete. Demographic and socio-economic questions are modified from demographic questions on the Behavioral Risk Factor Surveillance System survey, administered by the Centers for Disease Control and Prevention.

Objectives
1.1 Objectives
The specific aim of this study is to assess the effectiveness of two different mailed outreach activities (sequential choice of colonoscopy then FIT, active choice of colonoscopy and FIT) versus colonoscopy only in increasing participation in CRC screening.

1.2 Primary Outcome Variable
The primary outcome is CRC screening completion (FIT or colonoscopy) within 4 months of initial outreach

1.3 Secondary Outcome Variable(s)
The secondary outcome is the choice of screening test between FIT and colonoscopy.

Additional outcomes include demographic and socioeconomic characteristics of subjects who participate, as well as exploratory qualitative data regarding experience with the different outreach methodologies. Additional outcome is the percentage of FIT screening results that are positive, percentage of those positive FIT tests that receive follow-up diagnostic colonoscopy, and percentage of colonoscopies that find adenomas, advanced adenomas, and cancer.

Background
1.1 Program Goals
Despite effective strategies for prevention, early detection, and treatment, colorectal cancer (CRC) is the third most common type of cancer and second leading cause of cancer death in the United States. The US Preventive Services Task Force (USPSTF) recommends routine CRC screening for all individuals aged 50-75; yet, despite aggressive public health efforts to promote screening, national rates are still suboptimal at 59-64%.

Colonoscopy is the predominant form of screening in this country due to perceived effectiveness by providers, but it entails a significant cost in time, resources, and perceived discomfort. The fecal

immunochemical test (FIT) is an attractive screening option as it is less invasive than traditional lower endoscopy and can be mailed to patients to complete at home. A recent study has shown that offering the choice of colonoscopy or stool-based testing in a clinic setting increases screening rates, but receipt of colonoscopy has better durability since stool-based testing has to occur every year.

In this study, we will be using population-based outreach screening to evaluate the feasibility of a proactive screening program to promote fecal immunochemical testing (FIT) and/or colonoscopy through mailed outreach by leveraging principles of behavioral economics. We know that mailed FIT outreach circumvents the need for an office visit and eliminates friction in the screening process, since patients can perform testing at home in minutes, but it is not clear how patients may respond to different choice architecture about FIT versus colonoscopy as they have historically been seen as competing, rather than complementary strategies. Behavioral economics suggests that choice architecture may also impact response based on how the intervention is designed. For example, offering the choice of colonoscopy with the mailed FIT kit may enhance participation (as compared to offering colonoscopy alone) since it makes the decision an active choice, where the patient is choosing between two options as opposed to the traditional opt-in approach. Offering mailed FIT after colonoscopy, as is currently the standard during in-office visits, may also increase participation. By evaluating the effectiveness of these alternative choice approaches, we will enhance the public health capacity and efficiency to increase CRC screening uptake and reduce preventable death from this disease.

Statistical Considerations

1.1 Power and sample size

Approximately 900 potentially eligible subjects will be identified via a data abstraction by Penn Data Store. Through other projects in primary care practices at Penn Medicine we found the accuracy rate of the EMR algorithm to be approximately 75%. As such, we anticipate we will have enough patients to enroll 423 subjects (and randomize 141 into each arm). We estimate a base return rate for the colonoscopy only (control) arm to be 5%, and we will consider a meaningful increase in response rate to be 10 percentage points for both the sequential choice and active choice arms as compared to control. This will be sufficient sample size to detect a 10 percentage point increase in response rate using a two-tailed chi-square test with 80% power and a 5% level of significance.

1.2 Data analysis

The primary outcome is CRC screening completion (FIT or colonoscopy) within 4 months of initial outreach. We will conduct a chi-square analysis using Stata to compare arms 2 and 3 to arm 1 separately using intent-to-treat protocol. We will also compare arms 2 and 3 as a secondary analysis. We will quantitatively analyze the choice of screening test and evaluate the survey results by study arm. As exploratory analyses, we will evaluate response by practice location, age, gender, race/ethnicity, and income at the level of zip code.

Analysis will be conducted by blinded members of the research team at least four months after the last FIT is mailed.

Study Design

1.1 Design

Randomized: Subjects will be randomly assigned Study ID numbers and then randomized to one of

three study arms stratified by practice location using a computer-generated randomization algorithm. The research coordinator will record the randomization assignments on a master list which will be maintained on a password protected computer in a locked office. The research coordinator and research assistants will assemble the mailings based on this master list.

Blinding: The investigators will be blinded to the randomization assignment. The research coordinator and research assistants will be unblinded. The blind may be broken for clinical care purposes.

<u>1.2 Consent Process</u>

Waiver of consent for the main portion of this pilot study is being requested, as this study involves no more than minimal risk to subjects. Colonoscopy and FIT are clinically available and utilized tests used to screen for colorectal cancer. The outreach methods in the three arms are all offered during routine clinical care either at Penn Medicine or other health systems across the country. The only research related activity is the randomization of subjects to different outreach strategies that would typically occur in practice. Subjects will receive information about the risks and benefits of FIT through our outreach and about the risk and benefits of colonoscopy from the physician performing the procedure.

Subjects rights and welfare will not be adversely affected by the waiver of authorization and consent. All subjects will have the opportunity to voluntarily participate in CRC screening. Arm 1 (Colonoscopy only) will receive screening by colonoscopy if they elect. Arm 2 (Sequential Choice) will receive CRC screening either by Colonoscopy or FIT if they elect. Arm 3 (Active Choice) will receive CRC screening either by Colonoscopy or FIT if they elect. Each arm has the opportunity to engage in CRC screening through routine care as well.

We believe that we would not be able to practically conduct the research without waiver of consent. If we had to obtain either written or verbal consent ahead of time, it would substantially limit our study population and it may alter their participation in the intervention. Thus, we would only learn about the response rate for patients who we were able to speak to for consent. This, would limit the generalizability to practice. Obtaining waiver of consent would allow us to avoid the potential selection/volunteer bias for inclusion of patients particularly interested in screening that can occur when consent is required. Since our main objective is to understand the potential influence varying outreach strategies on subject behavior, we believe that obtaining consent would compromise our primary objective. Additionally, we have received waiver of consent for similar studies related to colorectal cancer screening outreach.

Verbal consent will be obtained from the subsample with whom we plan to conduct post intervention interviews (see script).

Study Duration

We anticipate conducting chart reviews for two months, mailed outreach and reminder follow-up for two months, waiting for completion of screening for an additional 4 months, and sub sample interview, data analysis and manuscript compilation for 4 months. Thus, we anticipate this pilot project to last 12 months. Project date of the proposed study: July 1, 2017 - June 30, 2018.

Resources Necessary for Human Research Protections

Shivan Mehta is the PI of this study. He is a gastroenterologist and assistant professor of medicine at the

Perelman School of Medicine. All members of the research team have completed CITI human subjects research training. The Research Coordinator will provide thorough education and training to the Research Assistants to ensure that they are well-prepared to carry out the duties in their job descriptions. Additionally, the Research Coordinator will audit 10% of the electronic medical record reviews that the Research Assistants complete, in order to check for compliance. Detailed Standard Operating Procedure documents for the project will be accessible to all members of the research team, which will keep research staff informed about the protocol and their related duties. There are adequate facilities to conduct the research; all research staff have adequate office space on the UPenn campus.

Target Population

Eligibility Criteria: The study population includes patients between 50 to 74 years old who have received care at the University City and Valley Forge CCA practices, are due for screening, and are asymptomatic for CRC.

Subjects Enrolled by Penn Researchers

423

Subjects Enrolled by Collaborating Researchers

0

Accrual

Approximately 900 potentially eligible subjects will be identified via a data abstraction by Penn Data Store. Through other projects in primary care practices at Penn Medicine we found the accuracy rate of the EMR algorithm to be approximately 75%. As such, we anticipate we will have enough patients to enroll 423 subjects (and randomize 141 into each arm). We estimate a base return rate for the colonoscopy only (control) arm to be 5%, and we will consider a meaningful increase in response rate to be 10 percentage points for both the sequential choice and active choice arms as compared to control.

This will be sufficient sample size to detect a 10 percentage point increase in response rate using a two-tailed chi-square test with 80% power and a 5% level of significance.

Key Inclusion Criteria

1. Between 50 and 74 years old
2. Has had at least two office visits at the University City or Valley Forge CCA practice within the past 2 years (at time of chart review)
3. Due for colorectal cancer (CRC) screening
4. Asymptomatic for CRC
5. Zip code listed in PennChart as part of the subjects address is within the Philadelphia-Wilmington-Camden Metropolitan Statistical Area
6. Has a primary care provider who is a University City or valley Forge Family Medicine provider

Key Exclusion Criteria

1. Has had prior colonoscopy within 10 years, sigmoidoscopy within 5 years, and FOBT/FIT within twelve months of the chart review (We will exclude patients who self-report undergoing any of the above procedures)
2. Has a history of CRC
3. Has a history of other GI cancer
4. Has history of confirmed Inflammatory Bowel Disease (IBD) (e.g. Crohns disease, ulcerative colitis) Irritable bowel syndrome does not exclude patients.
5. Has history of colitis other than Crohns disease or ulcerative colitis
6. Has had a colectomy
7. Has a relative that has been diagnosed with CRC
8. Has been diagnosed with Lynch Syndrome (i.e. HNPCC)
9. Has been diagnosed with Familial Adenomatous Polyposis (FAP)
10. Has iron deficiency anemia
11. Has history of lower GI bleeding
12. Has metastatic (Stage IV) blood or solid tumor cancer
13. Has end stage renal disease
14. Has had congestive heart failure
15. Has dementia
16. Has liver cirrhosis
17. Has any other condition that, in the opinion of the investigator, excludes the patient from participating in this study

Vulnerable Populations

No vulnerable populations are included in the research study.

Populations Vulnerable to Undue Influence or Coercion

We are not specifically targeting any vulnerable populations.

Subject Recruitment

900 potentially eligible subjects will be identified via a data abstraction by Penn Data Store using a data query algorithm that identifies patients who meet the inclusion criteria. Chart review will be conducted to source 423 eligible participants from this pool of subjects.

Subject Compensation

Participants will not be financially compensated for their participation.

Procedures

Screening - Phase 1: We will submit a data request of patients from the CCA practices of University City and Valley Forge from the Penn DataStore, based on an EMR algorithm that determines guideline-concordant colorectal cancer screening within PennChart. We estimate approximately 900 screen-eligible

patients will be identified through this EMR query, 423 of whom will be enrolled and randomized into the three arms. We anticipate conducting chart reviews for two months. During these chart reviews, the Research Assistants and the Research Coordinator will review the electronic medical record charts in PennChart (EPIC) to review each of the eligible patients pulled from Penn Data Store to confirm study eligibility.

Randomization - Phase 2: Subjects will be randomly assigned Study ID numbers and then randomized with stratification to one of three arms using a computer-generated randomization algorithm. The research coordinator will record the randomization assignments on a master list which will be maintained by the research coordinator on a password protected computer in a locked office. The research coordinator and research assistants will assemble the mailings based on this master list.

Outreach & Follow-up - Phase 3: Initial outreach letters will be sent to subjects. In the control arm the outreach will include a phone number to the VIP colonoscopy scheduling center. For the sequential choice arm, subjects will receive the same message as those in the control arm, indicating they are overdue for screening and including a phone number to the VIP screening scheduling center. For the active choice arm, subjects will receive both a mailed FIT kit and the option to call and schedule colonoscopy at the same time. After four weeks, a reminder letter will be sent to all subjects in all three arms who did not either schedule a colonoscopy appointment or complete and return a FIT kit. Subjects in the Control arm will receive a reminder letter that includes the same phone number to schedule colonoscopy. Subjects in the sequential choice arm will receive a second letter containing the phone number to the VIP screening scheduling center as well as a FIT kit. The active choice arm will receive a reminder that includes both the phone number to the VIP screening scheduling center and a reminder that they may alternatively complete the FIT that was initially mailed. The Research Coordinator and Research Assistants will be responsible for assembling the mailings. FIT kits will include a tube in which to deposit the stool sample, directions on how to collect and mail the sample, a letter about CRC screening, a lab requisition form, and a pre-paid return envelope.

Sub-sample Questionnaire - Phase 5:
A random subsample of 90 subjects will be selected to complete a questionnaire at least 4 months after they received the mailing. Through this questionnaire, the subjects will be asked to confirm their eligibility (e.g. that they had not had CRC screening within the USPSTF CRC screening guidelines) and provide additional demographic and socioeconomic status information as well as qualitative experience with the outreach materials and approach so that we can better understand what populations,
if any, may be more likely impacted by different types of outreach modalities when engaging in CRC screening. We anticipate these questionnaires to take 10 minutes to complete over the phone. The research staff will make no more than three attempts to speak directly with the subject.

Analysis Plan

<u>1.1 Power and Sample Size</u>

Approximately 900 potentially eligible subjects will be identified via a data abstraction by Penn Data Store. Through other projects in primary care practices at Penn Medicine we found the accuracy rate of the EMR algorithm to be approximately 75%. As such, we anticipate we will have enough patients to enroll 423 subjects (and randomize 141 into each arm). We estimate a base return rate for the colonoscopy only (control) arm to be 5%, and we will consider a meaningful increase in response rate to be 10 percentage points for both the sequential choice and active choice arms as compared to control.

This will be sufficient sample size to detect a 10 percentage point increase in response rate using a two-tailed chi-square test with 80% power and a 5% level of significance.

<u>1.2 Data analysis</u>

The primary outcome is CRC screening completion (FIT or colonoscopy) within 4 months of initial outreach. We will conduct a chi-square analysis using Stata to compare arms 2 and 3 to arm 1 separately using intent-to-treat protocol. We will also compare arms 2 and 3 as a secondary analysis. We will quantitatively analyze the choice of screening test and evaluate the survey results by study arm. As exploratory analyses, we will evaluate response by practice location, age, gender, race/ethnicity, and income at the level of zip code.

Analysis will be conducted by blinded members of the research team at least four months after the last FIT is mailed.

Data Confidentiality

Paper-based records will be kept in a secure location and only be accessible to personnel involved in the study. Computer-based files will only be made available to personnel involved in the study through the use of access privileges and passwords. Prior to access to any study- related information, personnel will be required to sign statements agreeing to protect the security and confidentiality of identifiable information. Wherever feasible, identifiers will be removed from study-related information. Precautions are in place to ensure the data is secure by using passwords and encryption, because the research involves web-based surveys.

Subject Confidentiality

Information about study subjects will be kept confidential and managed according to the requirements of the Health Insurance Portability and Accountability Act of 1996 (HIPAA). All PHI will be maintained on UPHS servers. Source documents are maintained in PennChart. No source documents will be printed or maintained in paper form at the study site. Data from PennChart will be recorded in Penn Medicine's REDCap system. The investigator and study team (which includes the research coordinator, and research assistants) will have access to PHI within PennChart and REDCap. We will label all PHI within REDCap as identifiable information so that de-identified exports are possible. All reports that include identifiable information will be stored on the Innovation Center secure drive, maintained behind the UPHS firewall. Once data analysis and manuscripts have been published, the databases will be removed from REDCap and the data will be de-identified on the secure drive. This deidentified dataset will be stored for up to five years after analysis is complete and manuscripts have been published. Once analysis is completed and any manuscripts are published, we will retain PHI no longer than seven years in accordance with government regulations, applicable policies, and institutional requirements.

Database Security/Protection Against Risk

To assure that patient, physician and other informant confidentiality is preserved, individual identifiers (such as name and medical record number/physician billing identifier) are stored in a single password protected system that is accessible only to study research, analysis and IT staff. This system is hosted on site at The University of Pennsylvania (UPenn) and is protected by a secure firewall. Once a participant is in this system, they will be given a unique study identification number (ID). Any datasets and computer

files that leave the firewall will be stripped of all identifiers and individuals will be referred to by their study ID. The study ID will also be used on all analytical files.

The initial patient information collected for screening and recruitment will consist of name, address, phone number, dates, medical records numbers and health plan account numbers. This information will come from Electronic Chart reviews.

Sensitive Research Information

This Research does not involve collection of sensitive information about the subjects that should be excluded from the electronic medical record.

Subject Privacy

We will only interact with the subsample of subjects with which we plan to call to conduct a follow-up questionnaire. With these subjects, we will conduct phone calls in a private area. When we call subjects, we will confirm the identify before administering the questionnaire. We will not be interacting with subjects in person.

Data Disclosure

FIT test results will be disclosed to the subject's primary care physician for continuity of care.

Protected Health Information/Data Protection

- Name
- Street address, city, county, precinct, zip code, and equivalent geocodes
- All elements of dates (except year) for dates directly related to an individual and all ages over 89
- Telephone and fax numbers
- Electronic mail addresses
- Medical record numbers
- Health Plan ID numbers

Consent Process

<u>1.1 Overview</u>

Waiver of consent for the main portion of this pilot study is being requested, as this study involves no more than minimal risk to subjects. Please see below. Verbal consent will be obtained from the subsample with whom we plan to conduct post intervention interviews (see script).

<u>1.2 Children and Adolescents</u>

Not applicable

<u>1.3 Adult Subjects Not Competent to Give Consent</u>

Waiver of consent is being requested.

Waiver of Consent

1.1 Minimal Risk

This study involves no more than minimal risk to subjects. Colonoscopy and FIT are clinically available and utilized tests used to screen for colorectal cancer. The outreach methods in the three arms are all offered during routine clinical care either at Penn Medicine or other health systems across the country. The only research related activity is the randomization of subjects to different outreach strategies that would typically occur in practice.

1.2 Impact on Subjects Rights and Welfare

Subjects rights and welfare will not be adversely affected by the waiver of authorization and consent. All subjects will have the opportunity to voluntarily participate in CRC screening. Arm 1 (Colonoscopy only) will receive screening by colonoscopy if they elect. Arm 2 (Sequential Choice) will receive CRC screening either by Colonoscopy or FIT if they elect. Arm 3 (Active Choice) will receive CRC screening either by Colonoscopy or FIT if they elect. Each arm has the opportunity to engage in CRC screening through routine care as well.

1.3 Waiver Essential to Research

We believe that we would not be able to practically conduct the research without waiver of consent. If we had to obtain either written or verbal consent ahead of time, it would substantially limit our study population and it may alter their participation in the intervention. Thus, we would only learn about the response rate for patients who we were able to speak to for consent. This, would limit the generalizability to practice. Obtaining waiver of consent would allow us to avoid the potential selection/volunteer bias for inclusion of patients particularly interested in screening that can occur when consent is required. Since our main objective is to understand the potential influence varying outreach strategies on subject behavior, we believe that obtaining consent would compromise our primary objective. Additionally, we have received waiver of consent for similar studies related to colorectal cancer screening outreach.

1.4 Additional Information to Subjects

Subjects will receive information about the risks and benefits of FIT through our outreach and about the risk and benefits of colonoscopy from the physician performing the procedure.

1.5 Written Statement of Research

No statement of research will be provided.

Potential Study Risks

The risks associated with this study are no more than minimal. There is the potential risk of breach of confidentiality. We will minimize this risk by using de-identified information whenever possible and by maintaining all identifiable information on a secure drive and/or in a HIPAA-compliant system (e.g. REDCap). There is also the risk of psychological harm associated with being screened for cancer. We will minimize this risk by communicating the results of the screening test to the subject in a timely fashion and facilitating the scheduling of diagnostic testing if the screening test is positive (as is usual practice for screening outreach programs).

Potential Study Benefits

If a participant completes colonoscopy or completes and returns the FIT, both of which are standard clinical care, the subjects will potentially benefit from participation by increasing the chances of

identifying colorectal cancer at an early stage. Information learned from this study may benefit society through a better understanding of how to effectively increase overall participation rates in CRC screening which could in turn reduce the rate of CRC mortality.

Data and Safety Monitoring

Safety will be monitored on an ongoing basis by the PI and the study team. The PI or designee will review the study charts to evaluate events at each subject interaction to ensure the grade, relationship to the study procedure, expectedness and the course of action for each subject is documented.

Risk/Benefit Assessment

The risks associated with this study are no more than minimal. Better knowledge of how to increase mailed screening could potentially address one of the major barriers of accessing care, i.e. having patients come in for clinical office visits. Additionally, FIT is less invasive than colonoscopy and, according to the USPSTF, considered equally effective if conducted once a year (as opposed to having a colonoscopy once every ten years). For these reasons and those outlined in the above benefits section, the Principal Investigator believes that the risks of participating in the study are outweighed by the potential benefits of participating in the study.

Final Protocol

****New changes from initial protocol notated in bold, parts removed from initial protocol notated in strikethrough**

Abstract
A 3-arm randomized trial assessing whether the rate of completion of colorectal cancer screening is increased when patients receive a sequential choice in screening options (colonoscopy followed by Fecal Immunochemical Testing (FIT)) or an active choice (FIT or colonoscopy offered together) versus colonoscopy alone. The targeted population is patients within the University City and Valley Forge Community Care Associates (CCA) practices at the University of Pennsylvania Health System.

Study Instruments
The primary endpoint being evaluated is the rate of participation in colorectal cancer screening between the two intervention arms (Sequential Choice, Active Choice) versus the control arm (colonoscopy only). The FIT is a well validated tool for colorectal cancer screening and is one of the screening modalities recommended by the USPSTF. The control arm will be sent a letter inviting them to schedule screening colonoscopy directly through the Gastroenterology Call Center. If not scheduled within 4 weeks, subjects will receive a mailed reminder to call to schedule. The Sequential Choice arm will be sent the same initial outreach as the control arm. If not scheduled within 4 weeks, subjects will receive a mailed reminder including the call center number as well as the option to complete a FIT kit mailed along with the reminder. The Active Choice arm of the study will be sent a letter offering the choice of Colonoscopy or completion of the FIT kit included with the initial mailing. If subjects in this group have not either scheduled colonoscopy nor completed FIT within 4 weeks, they will receive a mailed reminder to call to schedule colonoscopy or to complete the original mailed FIT.

A sub-sample of 90 subjects will be called to complete a questionnaire over the phone ~~4~~ **beginning approximately 6** months after initial outreach was mailed. ~~The subjects will be asked to confirm their eligibility (e.g. that they had not had CRC screening within the USPSTF CRC screening guidelines) and provide additional demographic and socioeconomic information so that we can better understand what populations, if any, may have differential response rates. We will also ask them~~ **The subjects will be asked to explain their experience with CRC screening prior to our initial outreach (November 2017), as well as** about their perception of the impact and design of CRC screening outreach. **Additional questions will probe for potential barriers to screening participation and ways to improve participation in the future.** ~~Demographic and socioeconomic questions are modified from demographic questions on the Behavioral Risk Factor Surveillance System survey, administered by the Centers for Disease Control and Prevention.~~ See attached sub-sample questionnaire.

Group Modifications
For subjects in the Control (Colonoscopy only) arm of the study, the post outreach phone questionnaires will not include questions regarding mailed FIT.

Method for Assigning Subjects to Groups

Subjects will be randomly assigned Study ID numbers and then randomized to one of three study arms stratified by the two practice locations (University City and Valley Forge) using a computer-generated randomization algorithm. The research coordinator will record the randomization assignments on a master list which will be maintained by the research coordinator on a password protected computer in a locked office. The research coordinator and research assistants will assemble the mailings based on this master list.

Administration of Surveys and/or Process

90 subjects will be randomly selected for the questionnaire. We anticipate the post outreach questionnaires to take 10 minutes to complete over the phone. The research staff will make no more than three attempts to speak directly with the subject. Based on a previous project where we reached about 50% of patients via phone call, we anticipate reaching approximately 45 subjects (15 in each arm) to complete this sub-sample questionnaire.

Administration of Surveys

All subjects will complete a baseline and post-intervention survey. The baseline survey will collect basic demographics and baseline medication adherence and blood pressure monitoring frequency and be conducted over the phone after the participant has been consented. The post-intervention survey will collect similar adherence and monitoring information, as well as qualitative data regarding patient perceptions about the interventions. The post-intervention survey will be completed at the in-person 4 month visit. These surveys should take no more than 15 minutes to complete. Demographic and socio-economic questions are modified from demographic questions on the Behavioral Risk Factor Surveillance System survey, administered by the Centers for Disease Control and Prevention.

Objectives

<u>1.4</u> <u>Objectives</u>

The specific aim of this study is to assess the effectiveness of two different mailed outreach activities (sequential choice of colonoscopy then FIT, active choice of colonoscopy and FIT) versus colonoscopy only in increasing participation in CRC screening.

<u>1.5</u> <u>Primary Outcome Variable</u>

The primary outcome is CRC screening completion (FIT or colonoscopy) within 4 months of initial outreach

<u>1.6</u> <u>Secondary Outcome Variable(s)</u>

The secondary outcome is the choice of screening test between FIT and colonoscopy.

Additional outcomes include demographic and socioeconomic characteristics of subjects who participate, as well as exploratory qualitative data regarding experience with the different outreach methodologies. Additional outcome is the percentage of FIT screening results that are positive, percentage of those positive FIT tests that receive follow-up diagnostic colonoscopy, and percentage of colonoscopies that find adenomas, advanced adenomas, and cancer.

Background

1.1 Program Goals

Despite effective strategies for prevention, early detection, and treatment, colorectal cancer (CRC) is the third most common type of cancer and second leading cause of cancer death in the United States. The US Preventive Services Task Force (USPSTF) recommends routine CRC screening for all individuals aged 50-75; yet, despite aggressive public health efforts to promote screening, national rates are still suboptimal at 59-64%.

Colonoscopy is the predominant form of screening in this country due to perceived effectiveness by providers, but it entails a significant cost in time, resources, and perceived discomfort. The fecal immunochemical test (FIT) is an attractive screening option as it is less invasive than traditional lower endoscopy and can be mailed to patients to complete at home. A recent study has shown that offering the choice of colonoscopy or stool-based testing in a clinic setting increases screening rates, but receipt of colonoscopy has better durability since stool-based testing has to occur every year.

In this study, we will be using population-based outreach screening to evaluate the feasibility of a proactive screening program to promote fecal immunochemical testing (FIT) and/or colonoscopy through mailed outreach by leveraging principles of behavioral economics. We know that mailed FIT outreach circumvents the need for an office visit and eliminates friction in the screening process, since patients can perform testing at home in minutes, but it is not clear how patients may respond to different choice architecture about FIT versus colonoscopy as they have historically been seen as competing, rather than complementary strategies. Behavioral economics suggests that choice architecture may also impact response based on how the intervention is designed. For example, offering the choice of colonoscopy with the mailed FIT kit may enhance participation (as compared to offering colonoscopy alone) since it makes the decision an active choice, where the patient is choosing between two options as opposed to the traditional opt-in approach. Offering mailed FIT after colonoscopy, as is currently the standard during in-office visits, may also increase participation. By evaluating the effectiveness of these alternative choice approaches, we will enhance the public health capacity and efficiency to increase CRC screening uptake and reduce preventable death from this disease.

Statistical Considerations

1.1 Power and sample size

Approximately 900 potentially eligible subjects will be identified via a data abstraction by Penn Data Store. Through other projects in primary care practices at Penn Medicine we found the accuracy rate of the EMR algorithm to be approximately 75%. As such, we anticipate we will have enough patients to enroll 423 subjects (and randomize 141 into each arm). We estimate a base return rate for the colonoscopy only (control) arm to be 5%, and we will consider a meaningful increase in response rate to be ~~10~~ **11** percentage points for both the sequential choice and active choice arms as compared to control. This will be sufficient sample size to detect a ~~10~~ **11** percentage point increase in response rate using a two-tailed chi-square test with 80% power and a ~~5% level of significance.~~ Type I error rate of .025, accounting for two pairwise comparisons with Bonferroni correction (.05/2 = .025).

1.2 Data analysis

The primary outcome is CRC screening completion (FIT or colonoscopy) within 4 months of initial outreach. We will conduct a chi-square analysis using Stata to compare arms 2 and 3 to arm 1 separately using intent-to-treat protocol. We will also compare arms 2 and 3 as a secondary analysis. We will

quantitatively analyze the choice of screening test and evaluate the survey results by study arm. As exploratory analyses, we will evaluate response by practice location, age, gender, race/ethnicity, and income at the level of zip code.

Analysis will be conducted by blinded members of the research team at least four months after the last FIT is mailed.

Study Design

1.1 Design

Randomized: Subjects will be randomly assigned Study ID numbers and then randomized to one of three study arms stratified by practice location using a computer-generated randomization algorithm. The research coordinator will record the randomization assignments on a master list which will be maintained on a password protected computer in a locked office. The research coordinator and research assistants will assemble the mailings based on this master list.

Blinding: The investigators will be blinded to the randomization assignment. The research coordinator and research assistants will be unblinded. The blind may be broken for clinical care purposes.

1.2 Consent Process

Waiver of consent for the main portion of this pilot study is being requested, as this study involves no more than minimal risk to subjects. Colonoscopy and FIT are clinically available and utilized tests used to screen for colorectal cancer. The outreach methods in the three arms are all offered during routine clinical care either at Penn Medicine or other health systems across the country. The only research related activity is the randomization of subjects to different outreach strategies that would typically occur in practice. Subjects will receive information about the risks and benefits of FIT through our outreach and about the
risk and benefits of colonoscopy from the physician performing the procedure.

Subjects rights and welfare will not be adversely affected by the waiver of authorization and consent. All subjects will have the opportunity to voluntarily participate in CRC screening. Arm 1 (Colonoscopy only) will receive screening by colonoscopy if they elect. Arm 2 (Sequential Choice) will receive CRC screening either by Colonoscopy or FIT if they elect. Arm 3 (Active Choice) will receive CRC screening either by Colonoscopy or FIT if they elect. Each arm has the opportunity to engage in CRC screening through routine care as well.

We believe that we would not be able to practically conduct the research without waiver of consent. If we had to obtain either written or verbal consent ahead of time, it would substantially limit our study population and it may alter their participation in the intervention. Thus, we would only learn about the response rate for patients who we were able to speak to for consent. This, would limit the generalizability to practice. Obtaining waiver of consent would allow us to avoid the potential selection/volunteer bias for inclusion of patients particularly interested in screening that can occur when consent is required. Since our main objective is to understand the potential influence varying outreach strategies on subject behavior, we believe that obtaining consent would compromise our primary objective. Additionally, we have received waiver of consent for similar studies related to colorectal cancer screening outreach.

Verbal consent will be obtained from the subsample with whom we plan to conduct post intervention interviews (see script).

Study Duration

We anticipate conducting chart reviews for two months, mailed outreach and reminder follow-up for two months, waiting for completion of screening for an additional 4 months, and sub sample interview, data analysis and manuscript compilation for 4 months. Thus, we anticipate this pilot project to last 12 months. Project date of the proposed study: July 1, 2017 - June 30, 2018.

Resources Necessary for Human Research Protections

Shivan Mehta is the PI of this study. He is a gastroenterologist and assistant professor of medicine at the Perelman School of Medicine. All members of the research team have completed CITI human subjects research training. The Research Coordinator will provide thorough education and training to the Research Assistants to ensure that they are well-prepared to carry out the duties in their job descriptions. Additionally, the Research Coordinator will audit 10% of the electronic medical record reviews that the Research Assistants complete, in order to check for compliance. Detailed Standard Operating Procedure documents for the project will be accessible to all members of the research team, which will keep research staff informed about the protocol and their related duties. There are adequate facilities to conduct the research; all research staff have adequate office space on the UPenn campus.

Target Population

Eligibility Criteria: The study population includes patients between 50 to 74 years old who have received care at the University City and Valley Forge CCA practices, are due for screening, and are asymptomatic for CRC.

Subjects Enrolled by Penn Researchers

423

Subjects Enrolled by Collaborating Researchers

0

Accrual

Approximately 900 potentially eligible subjects will be identified via a data abstraction by Penn Data Store. Through other projects in primary care practices at Penn Medicine we found the accuracy rate of the EMR algorithm to be approximately 75%. As such, we anticipate we will have enough patients to enroll 423 subjects (and randomize 141 into each arm). We estimate a base return rate for the colonoscopy only (control) arm to be 5%, and we will consider a meaningful increase in response rate to be 10 percentage points for both the sequential choice and active choice arms as compared to control.

This will be sufficient sample size to detect a 10 percentage point increase in response rate using a two-tailed chi-square test with 80% power and a 5% level of significance.

Key Inclusion Criteria

1. Between 50 and 74 years old
2. Has had at least two office visits at the University City or Valley Forge CCA practice within the past 2 years (at time of chart review)
3. Due for colorectal cancer (CRC) screening
4. Asymptomatic for CRC
5. Zip code listed in PennChart as part of the subjects address is within the Philadelphia-Wilmington-Camden Metropolitan Statistical Area
6. Has a primary care provider who is a University City or valley Forge Family Medicine provider

Key Exclusion Criteria

1. Has had prior colonoscopy within 10 years, sigmoidoscopy within 5 years, and FOBT/FIT within twelve months of the chart review (We will exclude patients who self-report undergoing any of the above procedures)
2. Has a history of CRC
3. Has a history of other GI cancer
4. Has history of confirmed Inflammatory Bowel Disease (IBD) (e.g. Crohns disease, ulcerative colitis) Irritable bowel syndrome does not exclude patients.
5. Has history of colitis other than Crohns disease or ulcerative colitis
6. Has had a colectomy
7. Has a relative that has been diagnosed with CRC
8. Has been diagnosed with Lynch Syndrome (i.e. HNPCC)
9. Has been diagnosed with Familial Adenomatous Polyposis (FAP)
10. Has iron deficiency anemia
11. Has history of lower GI bleeding
12. Has metastatic (Stage IV) blood or solid tumor cancer
13. Has end stage renal disease
14. Has had congestive heart failure
15. Has dementia
16. Has liver cirrhosis
17. Has any other condition that, in the opinion of the investigator, excludes the patient from participating in this study

Vulnerable Populations

No vulnerable populations are included in the research study.

Populations Vulnerable to Undue Influence or Coercion

We are not specifically targeting any vulnerable populations.

Subject Recruitment

900 potentially eligible subjects will be identified via a data abstraction by Penn Data Store using a data query algorithm that identifies patients who meet the inclusion criteria. Chart review will be conducted

to source 423 eligible participants from this pool of subjects.

Subject Compensation

Participants will not be financially compensated for their participation.

Procedures

Screening - Phase 1: We will submit a data request of patients from the CCA practices of University City and Valley Forge from the Penn DataStore, based on an EMR algorithm that determines guideline-concordant colorectal cancer screening within PennChart. We estimate approximately 900 screen-eligible patients will be identified through this EMR query, 423 of whom will be enrolled and randomized into the three arms. We anticipate conducting chart reviews for two months. During these chart reviews, the Research Assistants and the Research Coordinator will review the electronic medical record charts in PennChart (EPIC) to review each of the eligible patients pulled from Penn Data Store to confirm study eligibility.

Randomization - Phase 2: Subjects will be randomly assigned Study ID numbers and then randomized with stratification to one of three arms using a computer-generated randomization algorithm. The research coordinator will record the randomization assignments on a master list which will be maintained by the research coordinator on a password protected computer in a locked office. The research coordinator and research assistants will assemble the mailings based on this master list.

Outreach & Follow-up - Phase 3: Initial outreach letters will be sent to subjects. In the control arm the outreach will include a phone number to the VIP colonoscopy scheduling center. For the sequential choice arm, subjects will receive the same message as those in the control arm, indicating they are overdue for screening and including a phone number to the VIP screening scheduling center. For the active choice arm, subjects will receive both a mailed FIT kit and the option to call and schedule colonoscopy at the same time. After four weeks, a reminder letter will be sent to all subjects in all three arms who did not either schedule a colonoscopy appointment or complete and return a FIT kit. Subjects in the Control arm will receive a reminder letter that includes the same phone number to schedule colonoscopy. Subjects in the sequential choice arm will receive a second letter containing the phone number to the VIP screening scheduling center as well as a FIT kit. The active choice arm will receive a reminder that includes both the phone number to the VIP screening scheduling center and a reminder that they may alternatively complete the FIT that was initially mailed. The Research Coordinator and Research Assistants will be responsible for assembling the mailings. FIT kits will include a tube in which to deposit the stool sample, directions on how to collect and mail the sample, a letter about CRC screening, a lab requisition form, and a pre-paid return envelope.

Sub-sample Questionnaire - Phase 5:

A random subsample of 90 subjects will be selected to complete a questionnaire at least 4 **approximately 6** months after they received the mailing. Through this questionnaire, ~~the~~ subjects **will be asked to explain their experience with CRC screening outreach. Additional questions will probe for potential barriers to screening participation, and ways to improve participation in the future.** ~~confirm their eligibility (e.g. that they had not had CRC screening within the USPSTF CRC screening guidelines) and provide additional demographic and socioeconomic status information as well as qualitative experience with the outreach materials and approach so that we can better understand what~~

~~populations, if any, may be more likely impacted by different types of outreach modalities when engaging in CRC screening.~~ We anticipate these questionnaires to take 10 minutes to complete over the phone. The research staff will make no more than three attempts to speak directly with the subject.

Analysis Plan

1.1 Power and Sample Size

Approximately 900 potentially eligible subjects will be identified via a data abstraction by Penn Data Store. Through other projects in primary care practices at Penn Medicine we found the accuracy rate of the EMR algorithm to be approximately 75%. As such, we anticipate we will have enough patients to enroll 423 subjects (and randomize 141 into each arm). We estimate a base return rate for the colonoscopy only (control) arm to be 5%, and we will consider a meaningful increase in response rate to be ~~10~~ **11** percentage points for both the sequential choice and active choice arms as compared to control. This will be sufficient sample size to detect a ~~10~~ **11** percentage point increase in response rate using a two-tailed chi-square test with 80% power and a **Type 1 error rate of .025, accounting for two pairwise comparisons with Bonferroni correction (.05/2 = .025).** ~~5% level of significance.~~

1.2 Data analysis

The primary outcome is CRC screening completion (FIT or colonoscopy) within 4 months of initial outreach. We will conduct a chi-square analysis using Stata to compare arms 2 and 3 to arm 1 separately using intent-to-treat protocol. We will also compare arms 2 and 3 as a secondary analysis. We will quantitatively analyze the choice of screening test and evaluate the survey results by study arm. As exploratory analyses, we will evaluate response by practice location, age, gender, race/ethnicity, and income at the level of zip code.

Analysis will be conducted by blinded members of the research team at least four months after the last FIT is mailed.

Data Confidentiality

Paper-based records will be kept in a secure location and only be accessible to personnel involved in the study. Computer-based files will only be made available to personnel involved in the study through the use of access privileges and passwords. Prior to access to any study- related information, personnel will be required to sign statements agreeing to protect the security and confidentiality of identifiable information. Wherever feasible, identifiers will be removed from study-related information. Precautions are in place to ensure the data is secure by using passwords and encryption, because the research involves web-based surveys.

Subject Confidentiality

Information about study subjects will be kept confidential and managed according to the requirements of the Health Insurance Portability and Accountability Act of 1996 (HIPAA). All PHI will be maintained on UPHS servers. Source documents are maintained in PennChart. No source documents will be printed or maintained in paper form at the study site. Data from PennChart will be recorded in Penn Medicine's REDCap system. The investigator and study team (which includes the research coordinator, and research assistants) will have access to PHI within PennChart and REDCap. We will label all PHI within REDCap as identifiable information so that de-identified exports are possible. All reports that include identifiable information will be stored on the Innovation Center secure drive,

maintained behind the UPHS firewall. Once data analysis and manuscripts have been published, the databases will be removed from REDCap and the data will be de-identified on the secure drive. This deidentified dataset will be stored for up to five years after analysis is complete and manuscripts have been published. Once analysis is completed and any manuscripts are published, we will retain PHI no longer than seven years in accordance with government regulations, applicable policies, and institutional requirements.

Database Security/Protection Against Risk

To assure that patient, physician and other informant confidentiality is preserved, individual identifiers (such as name and medical record number/physician billing identifier) are stored in a single password protected system that is accessible only to study research, analysis and IT staff. This system is hosted on site at The University of Pennsylvania (UPenn) and is protected by a secure firewall. Once a participant is in this system, they will be given a unique study identification number (ID). Any datasets and computer files that leave the firewall will be stripped of all identifiers and individuals will be referred to by their study ID. The study ID will also be used on all analytical files.

The initial patient information collected for screening and recruitment will consist of name, address, phone number, dates, medical records numbers and health plan account numbers. This information will come from Electronic Chart reviews.

Sensitive Research Information

This Research does not involve collection of sensitive information about the subjects that should be excluded from the electronic medical record.

Subject Privacy

We will only interact with the subsample of subjects with which we plan to call to conduct a follow-up questionnaire. With these subjects, we will conduct phone calls in a private area. When we call subjects, we will confirm the identify before administering the questionnaire. We will not be interacting with subjects in person.

Data Disclosure

FIT test results will be disclosed to the subject's primary care physician for continuity of care.

Protected Health Information/Data Protection

- Name
- Street address, city, county, precinct, zip code, and equivalent geocodes
- All elements of dates (except year) for dates directly related to an individual and all ages over 89
- Telephone and fax numbers
- Electronic mail addresses
- Medical record numbers
- Health Plan ID numbers

Consent Process

1.1 Overview

Waiver of consent for the main portion of this pilot study is being requested, as this study involves no more than minimal risk to subjects. Please see below. Verbal consent will be obtained from the subsample with whom we plan to conduct post intervention interviews (see script).

1.2 Children and Adolescents

Not applicable

1.3 Adult Subjects Not Competent to Give Consent

Waiver of consent is being requested.

Waiver of Consent

1.1 Minimal Risk

This study involves no more than minimal risk to subjects. Colonoscopy and FIT are clinically available and utilized tests used to screen for colorectal cancer. The outreach methods in the three arms are all offered during routine clinical care either at Penn Medicine or other health systems across the country. The only research related activity is the randomization of subjects to different outreach strategies that would typically occur in practice.

1.2 Impact on Subjects Rights and Welfare

Subjects rights and welfare will not be adversely affected by the waiver of authorization and consent. All subjects will have the opportunity to voluntarily participate in CRC screening. Arm 1 (Colonoscopy only) will receive screening by colonoscopy if they elect. Arm 2 (Sequential Choice) will receive CRC screening either by Colonoscopy or FIT if they elect. Arm 3 (Active Choice) will receive CRC screening either by Colonoscopy or FIT if they elect. Each arm has the opportunity to engage in CRC screening through routine care as well.

1.3 Waiver Essential to Research

We believe that we would not be able to practically conduct the research without waiver of consent. If we had to obtain either written or verbal consent ahead of time, it would substantially limit our study population and it may alter their participation in the intervention. Thus, we would only learn about the response rate for patients who we were able to speak to for consent. This, would limit the generalizability to practice. Obtaining waiver of consent would allow us to avoid the potential selection/volunteer bias for inclusion of patients particularly interested in screening that can occur when consent is required. Since our main objective is to understand the potential influence varying outreach strategies on subject behavior, we believe that obtaining consent would compromise our primary objective. Additionally, we have received waiver of consent for similar studies related to colorectal cancer screening outreach.

1.4 Additional Information to Subjects

Subjects will receive information about the risks and benefits of FIT through our outreach and about the risk and benefits of colonoscopy from the physician performing the procedure.

1.5 Written Statement of Research
No statement of research will be provided.

Potential Study Risks
The risks associated with this study are no more than minimal. There is the potential risk of breach of confidentiality. We will minimize this risk by using de-identified information whenever possible and by maintaining all identifiable information on a secure drive and/or in a HIPAA-compliant system (e.g. REDCap). There is also the risk of psychological harm associated with being screened for cancer. We will minimize this risk by communicating the results of the screening test to the subject in a timely fashion and facilitating the scheduling of diagnostic testing if the screening test is positive (as is usual practice for screening outreach programs).

Potential Study Benefits
If a participant completes colonoscopy or completes and returns the FIT, both of which are standard clinical care, the subjects will potentially benefit from participation by increasing the chances of identifying colorectal cancer at an early stage. Information learned from this study may benefit society through a better understanding of how to effectively increase overall participation rates in CRC screening which could in turn reduce the rate of CRC mortality.

Data and Safety Monitoring
Safety will be monitored on an ongoing basis by the PI and the study team. The PI or designee will review the study charts to evaluate events at each subject interaction to ensure the grade, relationship to the study procedure, expectedness and the course of action for each subject is documented.

Risk/Benefit Assessment
The risks associated with this study are no more than minimal. Better knowledge of how to increase mailed screening could potentially address one of the major barriers of accessing care, i.e. having patients come in for clinical office visits. Additionally, FIT is less invasive than colonoscopy and, according to the USPSTF, considered equally effective if conducted once a year (as opposed to having a colonoscopy once every ten years). For these reasons and those outlined in the above benefits section, the Principal Investigator believes that the risks of participating in the study are outweighed by the potential benefits of participating in the study.

Summary of Protocol Changes Modifications LOG

Protocol: Choice Architecture and Colorectal Cancer Screening Outreach
University of Pennsylvania Principal Investigator: Shivan Mehta, MD

Date of Submission	Description of Modification	Rationale for Modification	Approval date
06/28/2017	Initial submission		07/28/2017
09/06/2017	Updates to study personnel	Add Research Assistant: Induru Vikrant	09/13/2017
11/21/2017	Updates to study personnel	Add student Research Assistant: Hoyt Gong<br>Remove student research assistant: Aaron Aahn	11/29/2017
02/06/2018	1) Updates to study personnel<br>2) Updated patient materials (questionnaire)	1) Remove student research assistant: Hoyt Gong<br>2) modified questionnaire to be more open ended, to gather more information and be more hypothesis generating than specific	02/12/2018
04/27/2018	1) Updated patient materials (questionnaire)<br>2) Updated study protocol (Phase 5: Sub-sample Questionnaire)	Revised the questionnaire to be more of a guided interview with open ended questions to gather a broader set of experiences, aimed at informing further research opportunities.	05/07/2018
05/16/2018	1) Updates to study personnel<br>2) Updated study protocol<br>3) Updated study protocol: analysis plan	1) Remove student research assistant: Tim McAuliffe; Remove study contact Rebecca Pepe; Add student research assistant: Humphrey Shen<br>2) Updated format to study protocol to reflect original and all modifications<br>3) Before beginning any analysis, we felt it necessary to revisit outcomes and provide a more detailed/thorough analysis plan to ensure both were as complete as possible.	05/16/2018
06/18/2018	1) Update study personnel<br>2) Continuing Review	1) Add student intern: David Santos	07/27/2018

Initial Statistical Analysis Plan

Analysis Plan

1.1 Power and Sample Size

Approximately 900 potentially eligible subjects will be identified via a data abstraction by Penn Data Store. Through other projects in primary care practices at Penn Medicine we found the accuracy rate of the EMR algorithm to be approximately 75%. As such, we anticipate we will have enough patients to enroll 423 subjects (and randomize 141 into each arm). We estimate a base return rate for the colonoscopy only (control) arm to be 5%, and we will consider a meaningful increase in response rate to be 10 percentage points for both the sequential choice and active choice arms as compared to control. This will be sufficient sample size to detect a 10 percentage point increase in response rate using a two-tailed chi-square test with 80% power and a 5% level of significance.

1.2. Data Analysis

The primary outcome is CRC screening completion (FIT or colonoscopy) within 4 months of initial outreach. We will conduct a chi-square analysis using Stata to compare arms 2 and 3 to arm 1 separately using intent-to-treat protocol. We will also compare arms 2 and 3 as a secondary analysis. We will quantitatively analyze the choice of screening test and evaluate the survey results by study arm. As exploratory analyses, we will evaluate response by practice location, age, gender, race/ethnicity, and income at the level of zip code.

Analysis will be conducted by blinded members of the research team at least four months after the last FIT is mailed.

Final Statistical Analysis Plan

****New changes from initial protocol notated in bold, parts removed from initial protocol notated in strikethrough**

Analysis Plan

1.1 Power and Sample Size

Approximately 900 potentially eligible subjects will be identified via a data abstraction by Penn Data Store. Through other projects in primary care practices at Penn Medicine we found the accuracy rate of the EMR algorithm to be approximately 75%. As such, we anticipate we will have enough patients to enroll 423 subjects (and randomize 141 into each arm). We estimate a base return rate for the colonoscopy only (control) arm to be 5%, and we will consider a meaningful increase in response rate to be ~~10~~ **11** percentage points for both the sequential choice and active choice arms as compared to control. This will be sufficient sample size to detect a ~~10~~ **11** percentage point increase in response rate using a two-tailed chi-square test with 80% power and a Type 1 error rate of .025, accounting for two pairwise comparisons with Bonferroni correction (.05/2 = .025). ~~5% level of significance.~~

1.2. Data Analysis

The primary outcome is CRC screening completion (FIT or colonoscopy) within 4 months of initial outreach. We will conduct a chi-square analysis using Stata to compare arms 2 and 3 to arm 1 separately using intent-to-treat protocol. We will also compare arms 2 and 3 as a secondary analysis. We will quantitatively analyze the choice of screening test and evaluate the survey results by study arm. As exploratory analyses, we will evaluate response by practice location, age, gender, race/ethnicity, and income at the level of zip code.

Analysis will be conducted by blinded members of the research team at least four months after the last FIT is mailed.

Summary of Statistical Analysis Plan Modifications

Before analysis, we felt it necessary to revisit outcomes and provide a more detailed/thorough analysis plan to ensure both were as complete as possible. The analysis plan was updated to be more descriptive of the specific comparisons that would be made, to include communication modality and demographics.

Appendix A – Final Survey Instrument

We're calling from <Penn Medicine Pt's Practice> to ask you a few questions about screening for colon cancer. The questions shouldn't take more than 10-15 minutes to complete, and we value your input on this important subject. There are no wrong answers and your responses could help us better care for our patients in the future.

Do you have a few minutes now to answer a few questions?

1) **What have you heard about getting screened for colon cancer? Whether you've had experience with it it firsthand or heard about it from someone else, anything you've heard is helpful to us.** (Probe for awareness of screening options, social influence, barriers, frequency, cost, ease, any past screening/non-screening experiences)

2) **Prior to November 2017, had you ever been screened for colon cancer before? Y/N**
   If N: question 3
   If Y: which test?
   If Y: do you remember when?
   If Y: do you remember where it was done?

3) **In November 2017, we mailed you an invitation to participate in colon cancer screening. What was your experience with that invitation?**
   (if no recollection/didn't receive it, skip to question 5)

4) Our records show that you didn't complete FIT/Colonoscopy after receiving that invitation. Don't worry – you're not alone, many people didn't**! Can you tell me about how that invitation helped you think about getting yourself screened for colon cancer? What are some of the reasons you decided not to get it done? What would facilitate you getting screened in the future?**

   or

   Our records show that you completed FIT/Colonoscopy after receiving that invitation – that's fantastic! **Can you tell me about how that invitation helped you think about getting yourself screened for colon cancer? What are some of the reasons you decided to get it done?**

   a. Follow-up: **Good or bad, we'd love to hear your screening story so we can capitalize on our strengths and improve where we fall short. Can you tell us about this colon cancer screening experience?** (Convenience/ease, prep, time it took, confidence in process/results, repeat, what would you tell others about it?)

5) The FIT test is an at-home screening test for colon cancer where you send a swab of stool to the lab to test for blood. Most doctors want their patients who are 50-75 years old to either do this test once per year, or get a colonoscopy once every ten years. **In the future, would you prefer to have a colonoscopy every 10 years or take a FIT every year?**

a) FIT
b) Colonoscopy
c) No preference
d) Prefer not to be screened

6) Can you tell us more about why FIT/Colonoscopy is your preference?

7) What would be the most helpful thing Penn Medicine could do to help ensure you get screened for colon cancer regularly?

Thank you for taking the time to give us your input, we appreciate it!

If you have any questions about this call, the FIT kit, colonoscopy or colon cancer screening in general, please call us at XXX-XXX-XXXX or speak with your Doctor.

Appendix B – Mailed Outreach Language

<u>Initial Outreach</u>

Colonoscopy Only

Our records show that you may be overdue for your colon cancer screening
Penn Medicine is offering a special VIP hotline to patients for scheduling screening colonoscopy.
Please call XXX-XXX-XXXX to schedule your colonoscopy right away.

Call the VIP hotline XXX-XXX-XXXX to schedule your screening colonoscopy today!

Sequential Choice

Our records show that you may be overdue for your colon cancer screening
Penn Medicine is offering a special VIP hotline to patients for scheduling screening colonoscopy.
Please call XXX-XXX-XXXX to schedule your colonoscopy right away.

Call the VIP hotline XXX-XXX-XXXX to schedule your screening colonoscopy!

Active Choice

Our records show that you may be overdue for your colon cancer screening
Penn Medicine is offering a special VIP hotline to patients for scheduling screening colonoscopy.
Please call XXX-XXX-XXXX to schedule your colonoscopy right away.
Alternatively, you may choose to instead complete and return the enclosed stool test called FIT (Fecal Immunochemical Test).

Call the VIP hotline XXX-XXX-XXXX to schedule your screening colonoscopy
-or-
complete and return your FIT today!

Reminder Outreach

Colonoscopy Only

Our records show that you may be overdue for your colon cancer screening
Penn Medicine is offering a special VIP hotline to patients for scheduling screening colonoscopy.
Please call XXX-XXX-XXXX to schedule your colonoscopy right away.

Call the VIP hotline XXX-XXX-XXXX to schedule your screening colonoscopy today!

Sequential Choice

Our records show that you may be overdue for your colon cancer screening
Penn Medicine is offering a special VIP hotline to patients for scheduling screening colonoscopy.
Please call XXX-XXX-XXXX to schedule your colonoscopy right away.
Alternatively, you may choose to instead complete and return the enclosed stool test called FIT (Fecal Immunochemical Test).

Call the VIP hotline XXX-XXX-XXXX to schedule your screening colonoscopy
-or-
complete and return your FIT today!

Active Choice

Our records show that you may be overdue for your colon cancer screening
Penn Medicine is offering a special VIP hotline to patients for scheduling screening colonoscopy.
Please call XXX-XXX-XXXX to schedule your colonoscopy right away.
Alternatively, you may choose to instead complete and return the enclosed stool test called FIT (Fecal Immunochemical Test).

Call the VIP hotline XXX-XXX-XXXX to schedule your screening colonoscopy
-or-
complete and return your FIT today!
