# Supplementary material for: Effect of Sequential or Active Choice for Colorectal Cancer Screening Outreach: A Randomized Clinical Trial
Source: JAMA Netw Open. 2019 Aug 30;2(8):e1910305. doi: 10.1001/jamanetworkopen.2019.10305 (PMC6724166; doi:10.1001/jamanetworkopen.2019.10305)
Supplement: Supplement 2. — Data Sharing Statement [file jamanetwopen-2-e1910305-s002.pdf]

# Data Sharing Statement

Mehta. Effect of Sequential or Active Choice for Colorectal Cancer Screening Outreach. *JAMA Netw Open*. Published August 30, 2019. 10.1001/jamanetworkopen.2019.10305

**Data**
**Data available:** No
